# Supplementary figures and images for: Huntington’s disease phenotypes are improved via mTORC1 modulation by small molecule therapy
Source: PLoS One. 2022 Aug 29;17(8):e0273710. doi: 10.1371/journal.pone.0273710 (PMC9423655; doi:10.1371/journal.pone.0273710)

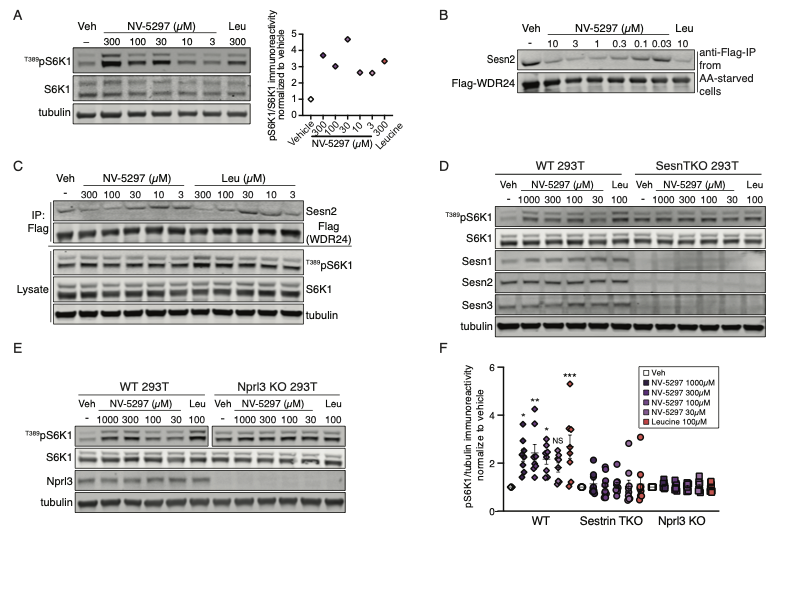

Supplement: S1 Fig — (A) Activation of the mTORC1 pathway by NV-5297. Immunoblot shows levels of phosphorylated S6K1 (T389pS6K1) and non-phosphorylated S6K1 in HEK-293T cells starved of leucine (Leu) for 50 minutes followed by addition of vehicle (Veh), NV-5297 or leucine for 10 minutes and the associated quantification. (B) NV-5297 disrupts Sestrin2/GATOR2 in the in vitro Sesn2/WDR24 protein-protein interaction assay in a dose-dependent manner. Immunoblotting of Sestrin 2 (Sesn2) after flag-WDR24 immunoprecipitation from amino acid-starved flag-WDR24 expressing HEK-293T cells followed by addition of NV-5297 or leucine at 10 μM for 10 minutes. (C) Dose-dependent activation of mTORC1 by NV-5297 or leucine correlates with disruption of Sesn2 from Flag-WDR24. Immunoblot shows levels of Sestrin2 bound to immunoprecipitated Flag-WDR24 and levels of T389pS6K1 in flag-WDR24 expressing HEK-293T cells were starved of leucine for 50 minutes followed by addition of vehicle, NV-5297 or leucine for 10 minutes. NV-5297 requires an intact Sestrins/GATOR pathway to mediate mTORC1 pathway activation. Representative immunoblots of T389pS6K1 and quantification of the normalized level of T389pS6K1 from 8 independent experiments (F) show TORC1 activity in unedited HEK-293T cells, HEK-293T cells deficient for Sestrin 1 (Sesn1), 2 (Sesn2) and 3 (Sesn3) (D) or HEK-293T cells deficient for the GATOR1 component Nprl3 (E) after 50 minutes leucine starvation followed by the addition of vehicle, NV-5297 or leucine for 10 minutes. Data are presented as mean ± SEM. *P ≤ 0.05, **P ≤ 0.01 and ***P ≤ 0.001 (One-way ANOVA with Tukey’s multiple comparison tests). (TIFF) [file pone.0273710.s001.tiff]

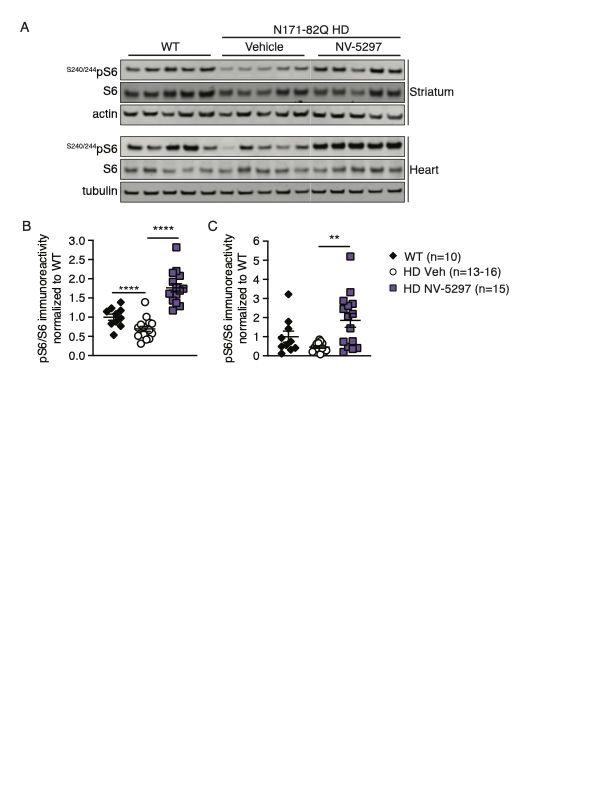

Supplement: S2 Fig — (A) Representative immunoblot for the phosphorylated (S240/244pS6), S6 and actin or tubulin in the striatum and heart of N171-82Q wild-type (WT) mice or N171-82Q HD mice respectively collected 1 hour after the last dose of a week of oral dosing with vehicle (Veh) or NV-5297 (160 mg/kg; A). Quantification of levels of S240/244pS6 in the striatum (B) and the heart (C) normalized to loading control levels and further normalized to untreated WT mice. Data are presented as mean ± SEM. **P ≤ 0.01, ***P ≤ 0.001 and ****P ≤ 0.0001 (Kruskal-Wallis test with Dunn’s multiple comparison tests). (TIFF) [file pone.0273710.s002.tiff]

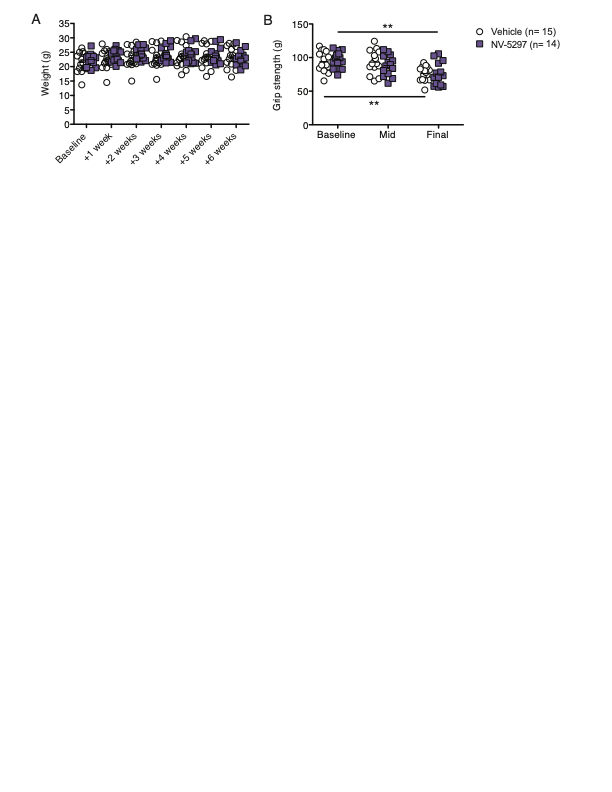

Supplement: S3 Fig — (A) Weight remained unaltered between N171-82Q male mice orally dosed with NV-5297 (160 mg/kg) for 6 weeks and vehicle treated males. (B) Forelimb grip strength also remained unaltered after 6 weeks of NV-5297 or vehicle-treated dosing. The forelimb grip strength decreased with time similarly in both groups. Data are presented as mean ± SEM. **P ≤ 0.01 (two-way repeated measure ANOVA followed by Tukey’s multiple comparisons test [A] and Dunnett’s multiple comparison tests [B]). (TIFF) [file pone.0273710.s003.tiff]

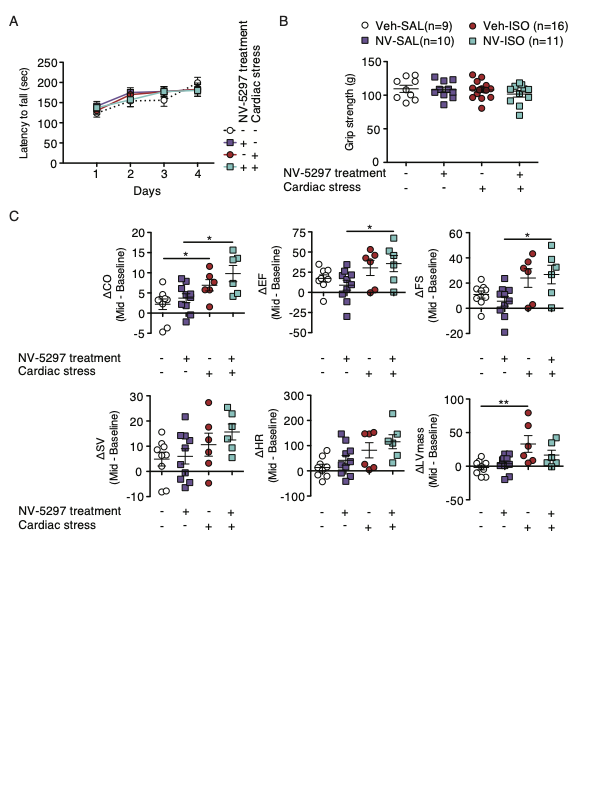

Supplement: S4 Fig — The baseline latency to fall from the accelerated rotarod (A) and the forelimb grip strength (B) was similar between the experimental groups. (C) N171-82Q male mice orally dosed daily with NV-5297 (160 mg/kg) or vehicle [Veh] starting at 6 weeks of age showed modifications in their heart function a week after implantation (at 10 weeks of age) of a saline [Sal] or isoprenaline [Iso] osmotic pump. CO: Cardiac output, EF: Ejection fraction, FS: Fractional shortening, SV: Stroke volume, HR: heart rate, LVmass: Left ventricular mass. (Veh-Sal n = 9, NV-Sal n = 10, Veh-Iso n = 6, NV-Iso n = 6). Data are presented as mean ± SEM. *P ≤ 0.05, **P ≤ 0.01 (Mixed-effects models [A, B] with Sidak’s multiple comparisons tests [C]). (TIFF) [file pone.0273710.s004.tiff]
