# Supplementary material for: Huntington’s disease phenotypes are improved via mTORC1 modulation by small molecule therapy
Source: PLoS One. 2022 Aug 29;17(8):e0273710. doi: 10.1371/journal.pone.0273710 (PMC9423655; doi:10.1371/journal.pone.0273710)
Supplement: S1 Table — CL: Clearance, F: Bioavailability, INF: Infinity, MRT: Mean residence time, OD: Oral dose, PK: Pharmacokinetics; Tmax: time to reach Cmax, Vss: Steady state volume of distribution. (PDF) [file pone.0273710.s005.pdf]

PK parameters of NV-5297 after an IV dose at 1 mg/kg		
PK parameters	Unit	Estimate
CL	L/hr/kg	0.8
V_{ss}	L/kg	1.26
Terminal $T_{1/2}$	hr	1.45
AUC_{last}	hr*ng/mL	1222
AUC_{INF}	hr*ng/mL	1245
MRT_{INF}	hr	1.56
**PK parameters of NV-5297 after an OD dose at 5 mg/kg**		
PK parameters	Unit	Estimate
Tmax	hr	0.5
C_{max}	Ng/mL	2893
Terminal $T_{1/2}$	hr	2.86
AUC_{last}	hr*ng/mL	6464
AUC_{INF}	hr*ng/mL	6471
F	%	104
