# Supplementary material for: Huntington’s disease phenotypes are improved via mTORC1 modulation by small molecule therapy
Source: PLoS One. 2022 Aug 29;17(8):e0273710. doi: 10.1371/journal.pone.0273710 (PMC9423655; doi:10.1371/journal.pone.0273710)
Supplement: S1 File — (PDF) [file pone.0273710.s006.pdf]

S1 File. Synthesis of NV-5297 [(S)-2-amino-5,5,5-trifluoro-4,4-dimethylpentanoic acid]

Synthetic scheme:

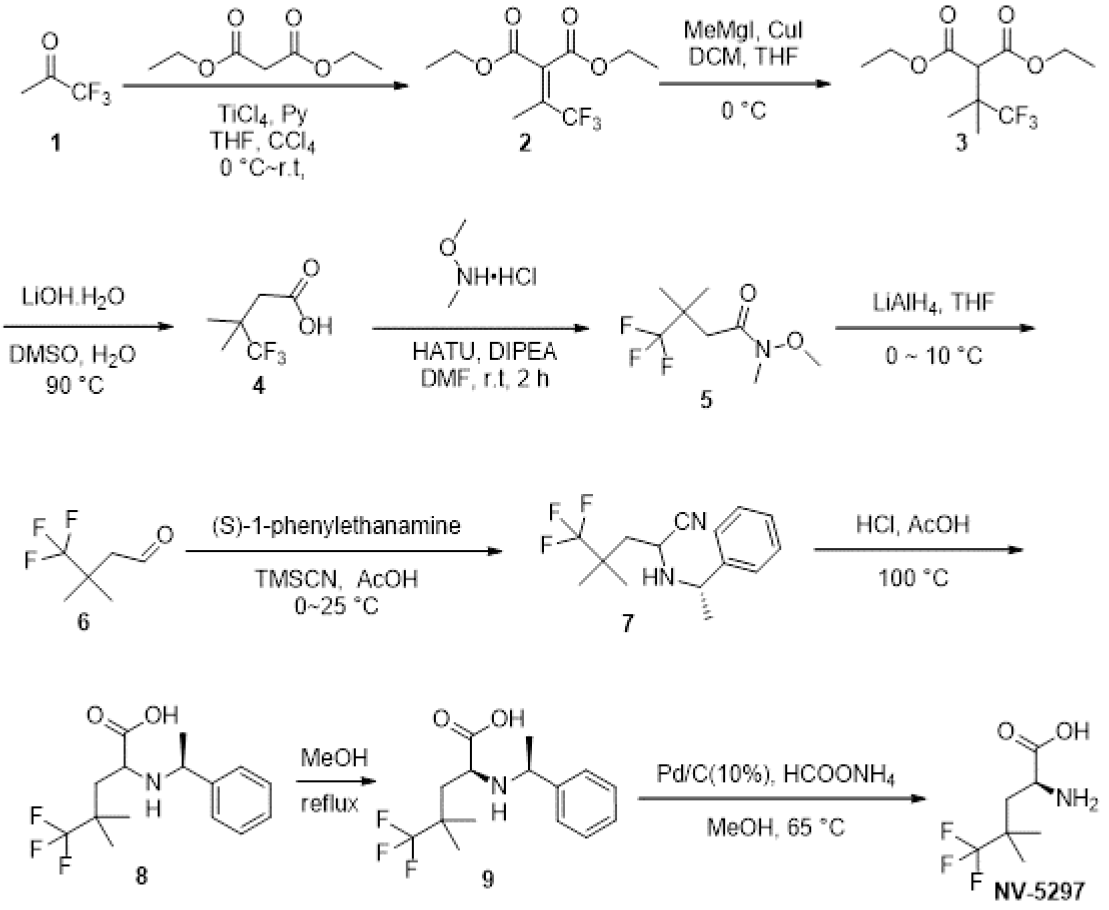

Procedures and characterization:

Step 1: diethyl 2-(1,1,1-trifluoropropan-2-ylidene)malonate:

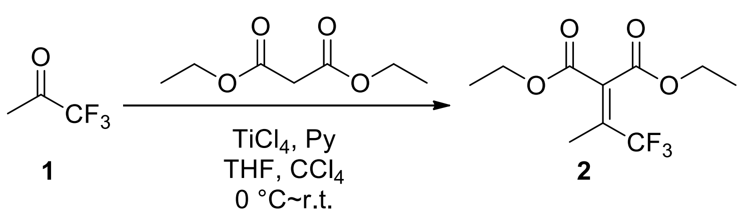

To the flask containing tetrahydrofuran (THF, 3.0 L) was added titanium tetrachloride (102 g, 538 mmol) in carbon tetrachloride (CCl_4; 104 g, 673 mmol) at 0 °C and the resulting reaction mixture

was stirred at the same temperature for 30 mins. Then 1,1,1-trifluoropropan-2-one (45.3 g, 404 mmol) and diethyl propanedioate (43.1 g, 269 mmol) were added dropwise at the same time. The resulting reaction mixture was stirred at the same temperature for one hour. Pyridine (106 g, 1345 mmol) was added and the reaction mixture was allowed to warm to 25 °C and stirred at the same temperature overnight. After completion of the reaction indicated by proton NMR (^{1}H-NMR), the reaction mixture was poured into water (1 L), filtered, and the filtrate was extracted with Methylene chloride (DCM, 1.0 L x 3). The organic phase was washed with brine (600 mL), dried (sodium sulfate [Na_2SO_4]), filtered and concentrated in vacuum to afford diethyl 2-(1,1,1-trifluoropropan-2-ylidene)malonate (68.3 g, 100%) as a yellow liquid.

^{1}H-NMR (400 MHz, deuterated chloroform [$CDCl_3$]): δ 4.29 (t, *J* = 7.2 Hz, 4H), 2.22 (s, 3H), 1.34-1.25 (m, 6H).

Step 2: diethyl 2-(1,1,1-trifluoro-2-methylpropan-2-yl)malonate:

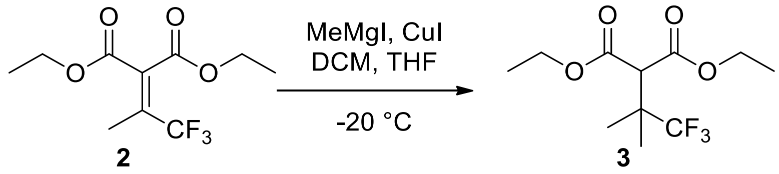

To a mixture of diethyl 2-(1,1,1-trifluoropropan-2-ylidene)malonate (60.0 g, 236 mmol) and copper iodide (CuI, 68 g, 354 mmol) in DCM (800 mL) and THF (200 mL) was added methylmagnesium iodide (MeMgI; 354 mL, 3M in Diethyl ether, 708 mmol) dropwise at -20 °C, and the reaction mixture was stirred at -20 ~ -10 °C for 1 h. After completion of the reaction indicated by liquid chromatography – mass spectroscopy (LCMS), the solution was poured into ice-water (1 L) and treated with saturated ammonium chloride (NH_4Cl) solution (500 mL), the mixture was stirred for 30 mins and filtered; the filtrate was extracted with DCM (500 mL); the organic phase was washed with water (400 mL x 2), and brine (400 mL), dried (Na_2SO_4), filtered

and concentrated in vacuum to afford diethyl 2-(1,1,1-trifluoro-2-methylpropan-2-yl) malonate (64 g, 100%) as a yellow liquid which was used for the next step.

^{1}H-NMR (400 MHz, $CDCl_3$): δ 4.22 (t, J = 6.8 Hz, 4H), 3.65 (s, 1H), 1.39 (s, 6H), 1.29-1.26 (m, 6H).

Step 3: 4,4,4-trifluoro-3,3-dimethylbutanoic acid:

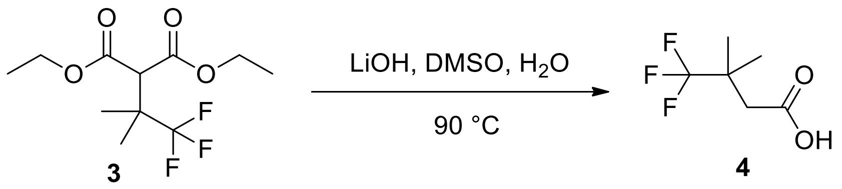

A mixture of diethyl 2-(1,1,1-trifluoro-2-methylpropan-2-yl)malonate (60.0 g, 222 mmol) and lithium hydroxide monohydrate (LiOH•H_2O, 47.0 g, 1111 mmol) in dimethyl sufoxide (DMSO, 500 mL) and water (H_2O, 50 mL) was heated to 90 °C overnight **(inner temperature must be lower than 100 °C).** After completion of the reaction indicated by LCMS, the mixture was diluted with water (2 L), extracted with PE stands for? (PE, 1000 mL), the aqueous phase was adjusted pH to 3-4 with 6M hydrochloric acid (HCl) in aqueous phase (aq.), extracted with DCM (500 mL×2), dried (Na_2SO_4), filtered and concentrated in vacuum to afford 4,4,4-trifluoro-3,3-dimethylbutanoic acid (41 g, crude) as a yellow liquid.

^{1}H-NMR (400 MHz, $CDCl_3$): δ 2.45 (s, 2 H), 1.23 (s, 6 H).

Step 4: 4,4,4-trifluoro-N-methoxy-N,3,3-trimethylbutanamide:

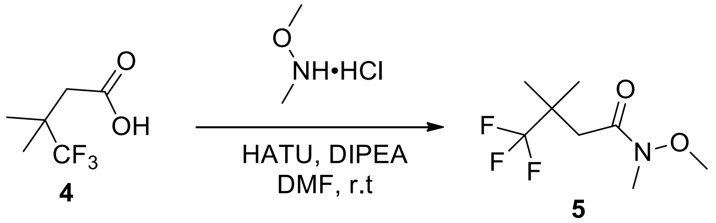

To a solution of 4,4,4-trifluoro-3,3-dimethylbutanoic acid (44.0 g, 258 mmol), N,O-dimethylhydroxylamine hydrochloride (50.0 g, 518 mol) and (1-[Bis(dimethylamino)methylene]-1H-1,2,3-triazolo[4,5-b]pyridinium 3-oxide hexafluorophosphate, Hexafluorophosphate Azabenzotriazole Tetramethyl Uronium) (HATU, 117 g, 310 mol) in dimethylformamide (DMF, 1 L) was added triethylamine (Et_3N, 130.0 g, 1290 mol) at 0 °C, and the reaction mixture was stirred room temperature for 17 h. After completion of the reaction indicated by LCMS, the mixture was diluted with water (3 L), extracted with diethyl ether (Et_2O, 1 L×3). The combined organic layers was washed with 1M HCl aq. (1 L×2), saturated sodium bicarbonate ($NaHCO_3$) aq. (1 L), water (1 L) and brine (1 L), dried (Na_2SO_4), filtered and concentrated in vacuum to afford 4,4,4-trifluoro-N-methoxy-N,3,3-trimethyl-butanamide (55 g, 100%, 2 steps) as a yellow liquid.

^{1}H-NMR (400 MHz, $CDCl_3$): δ 3.62 (s, 3H), 3.12 (s, 3H), 2.53 (s, 2H) , 1.22 (s, 6H).

Step 5: 4,4,4-trifluoro-3,3-dimethylbutanal:

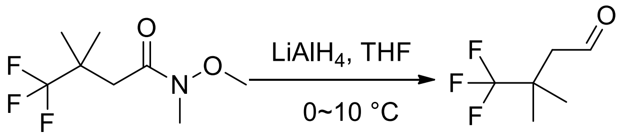

5 **6**

To a solution of 4,4,4-trifluoro-N-methoxy-N,3,3-trimethylbutanamide (40.0 g, 187 mmol) in THF (400 mL) was added lithium aluminum hydride ($LiAlH_4$, 7.13 g, 187 mmol) portion wise at 0 °C, and the reaction mixture was stirred at 0 °C for 1 h. After completion of the reaction indicated by LCMS, the reaction mixture was quenched by adding sodium sulfate decahydrate (Na_2SO_4•$10H_2O$), stirred at room temperature for 1 h, filtered and washed with Et_2O (1 L). The filtrate was used directly in the next step reaction without further purification.

Step 6: 5,5,5-trifluoro-4,4-dimethyl-2-(((S)-1-phenylethyl)amino)pentanenitrile:

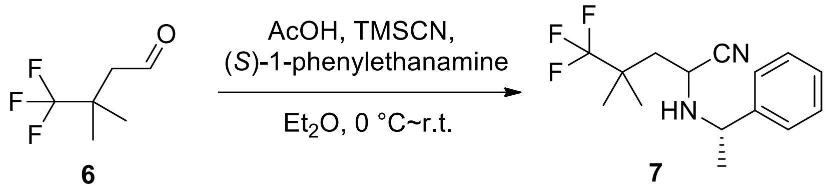

6 7

To the above solution was added (S)-1-phenylethanamine (40 mL), acetic acid (AcOH, 40 mL) and then trimethylsilyl cyanide (TMSCN, 40 mL) with ice-bath, and the solution was stirred at room temperature for 17 h. After completion of the reaction indicated by LCMS, the mixture diluted with EA stands for? (500 mL), washed with H_2O (500 mL x 2) and then concentrated to afford 5,5,5-trifluoro-4,4-dimethyl-2-(((S)-1-phenylethyl)amino) pentanenitrile (50 g, crude) as a brown liquid, which was used directly in the next step reaction without further purification. Electrospray isonization – mass spectroscopy (ESI-MS; EI^+, m/z): 285.3 $[M+H]^+$.

Step 7: (S)-5,5,5-trifluoro-4,4-dimethyl-2-(((S)-1-phenylethyl)amino)pentanoic acid:

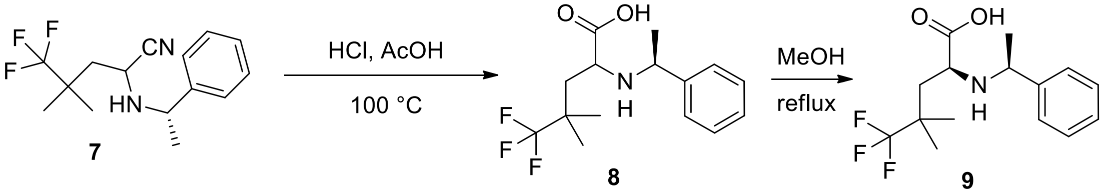

7 8 9

A solution of 5,5,5-trifluoro-4,4-dimethyl-2-(((S)-1-phenylethyl)amino)pentanenitrile (50 g, crude) in concentrated HCl (500 mL) and AcOH (100 mL) was heated to 100 °C for 64 h, After completion of the reaction indicated by LCMS, the mixture was concentrated to remove the solvent, adjusted pH to 12 with 1M sodium hydroxide (NaOH) aquous, extracted with PE stands for (200 mL). The aqueous phase was adjusted pH to 5-6 with 6M HCl aqueous, the formed

white solid was filtered, washed with water (50 mL) and dried in vacuum to obtain 9.5 g of white solid. The solid was suspended in methanol (MeOH, 750 mL), heated to reflux overnight and filtered in heat. The solid washed with MeOH and dried in vacuum to afford (S)-5,5,5-trifluoro-4,4-dimethyl-2-(((S)-1-phenylethyl)amino) pentanoic acid (4.5 g, 8.5%) as white solid.

^{1}H-NMR (400 MHz, DMSO-d_6): δ 7.47 (s, 5 H), 4.32 (d, *J* = 7.2 Hz, 1H), 3.18 (d, *J* = 7.2 Hz, 1H), 2.14-2.07 (m, 1H), 1.86-1.81 (m, 1H), 1.68 (d, *J* = 6.8 Hz, 3H), 1.11 (s, 3H), 0.98 (s, 3H).

Step 8: (S)-2-amino-5,5,5-trifluoro-4,4-dimethylpentanoic acid:

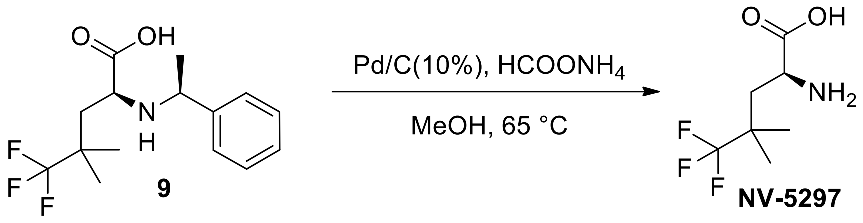

A mixture of (S)-5,5,5-trifluoro-4,4-dimethyl-2-(((S)-1-phenylethyl)amino)pentanoic acid (10 g, 33 mmol), ammonium formate ($HCOONH_4$; 10.4 g, 165 mmol) and palladium on carbon (Pd/C; 10%, 2.0 g) in MeOH (200 mL) was heated to 60 °C for 2 h with a balloon buffer, After completion of the reaction indicated by LCMS, the mixture was filtered through Celite, and the filtrate was concentrated to give the residual solid, which was wash with cold MeOH to afford (S)-2-amino-5,5,5-trifluoro-4,4-dimethylpentanoic acid (5 g, 76 %) as a white solid.

ESI-MS (EI^+, m/z): 200.0$[M+H]^+$.

^{1}H-NMR (400 MHz, DMSO-d_6): δ 3.63(t, *J* = 6.0 Hz, 1H), 2.35 (dd, *J* = 14.8, 5.2 Hz, 1H), 1.90 (dd, *J* = 14.8, 6.8 Hz, 1H), 1.26 (d, *J* = 3.2 Hz, 6H).
