# Supplementary material for: Huntington’s disease phenotypes are improved via mTORC1 modulation by small molecule therapy
Source: PLoS One. 2022 Aug 29;17(8):e0273710. doi: 10.1371/journal.pone.0273710 (PMC9423655; doi:10.1371/journal.pone.0273710)

Figure 1B

MWM: Molecular weight marker
5 Vehicle and 5 NV-5297 samples per blot.
All blots imaged with LI-COR imaging system.

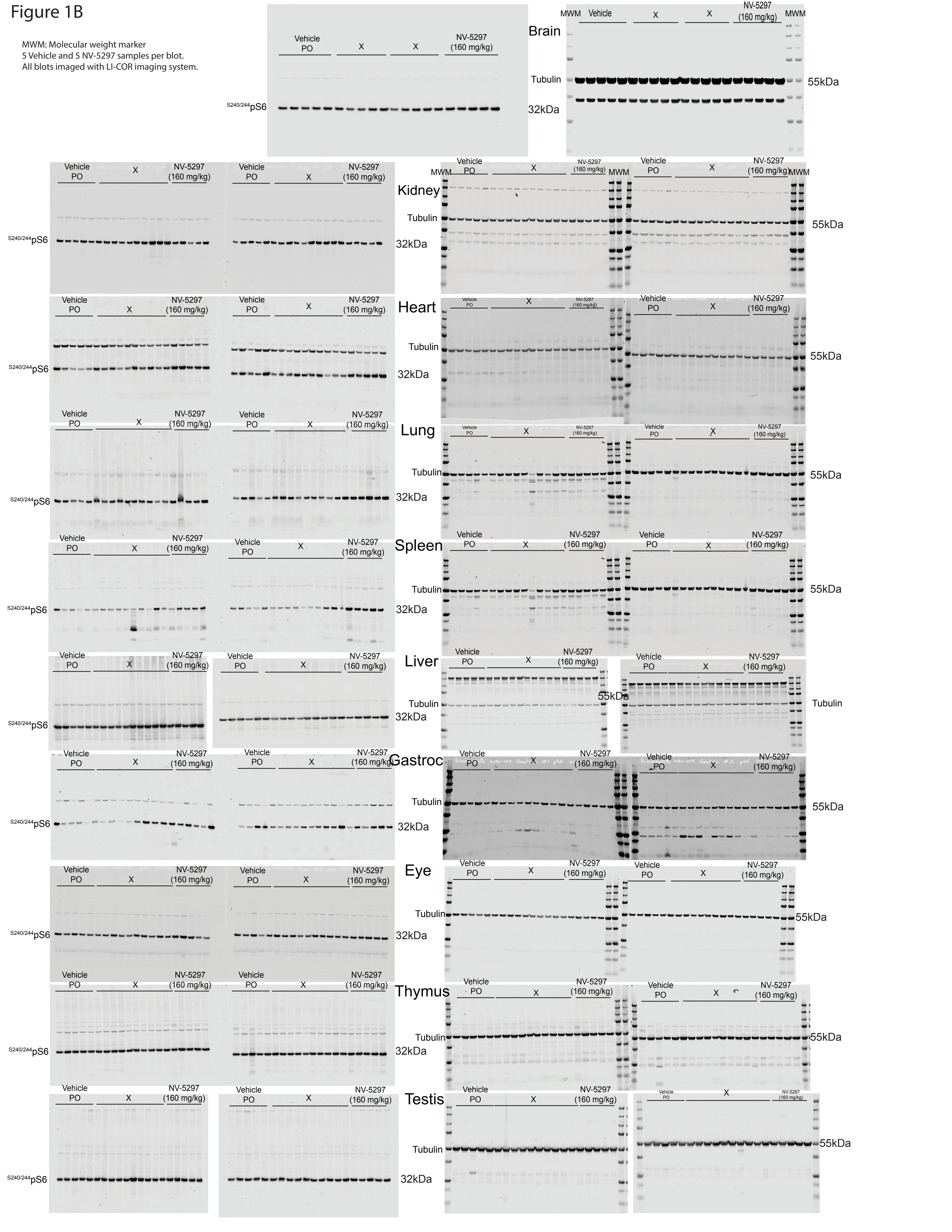

Figure 1D

MWM: Molecular weight markers
All blots imaged with LI-COR imaging system.

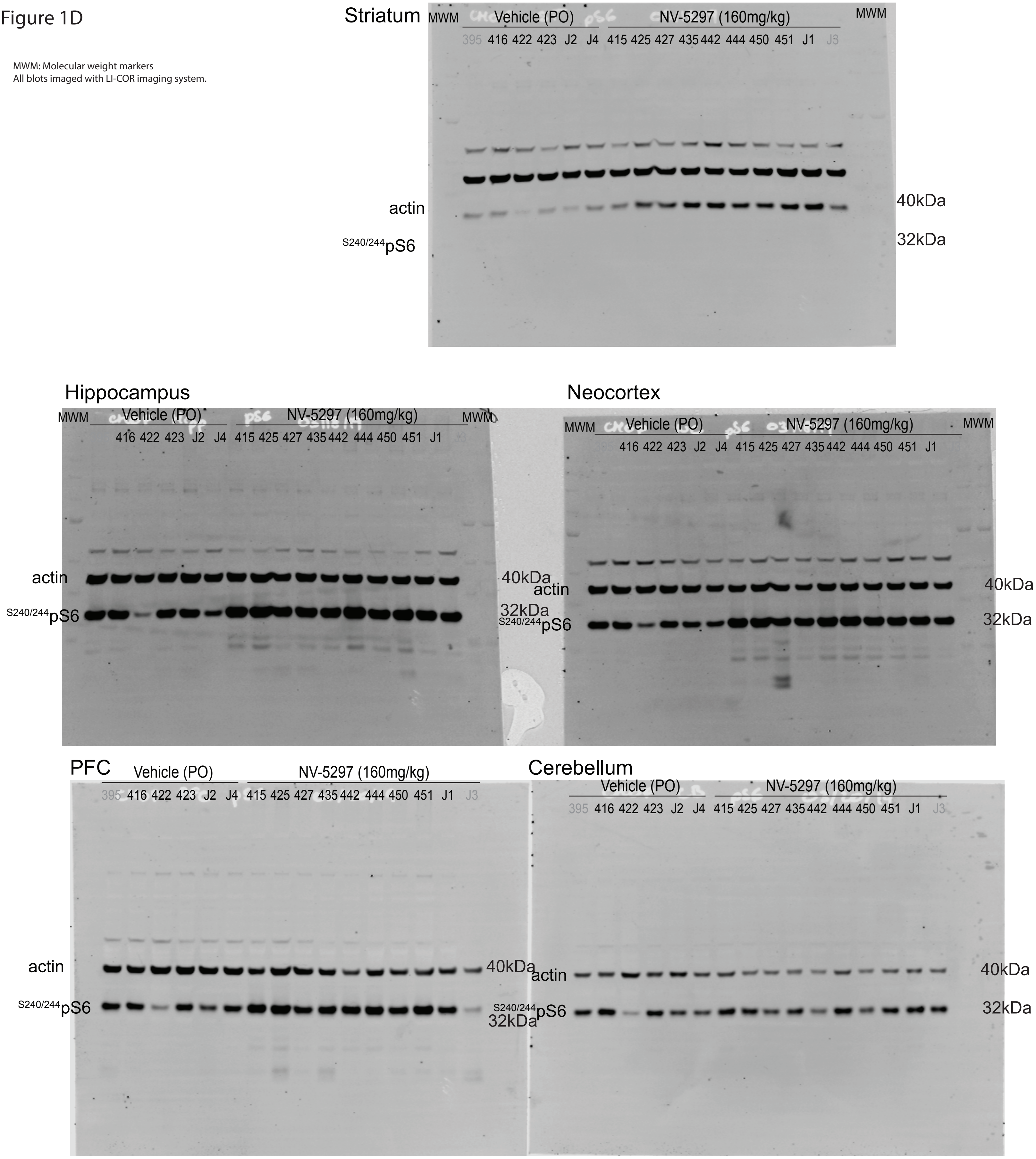

Figure 3A

MWM: Molecular weight marker

All blots imaged with LI-COR imaging system.

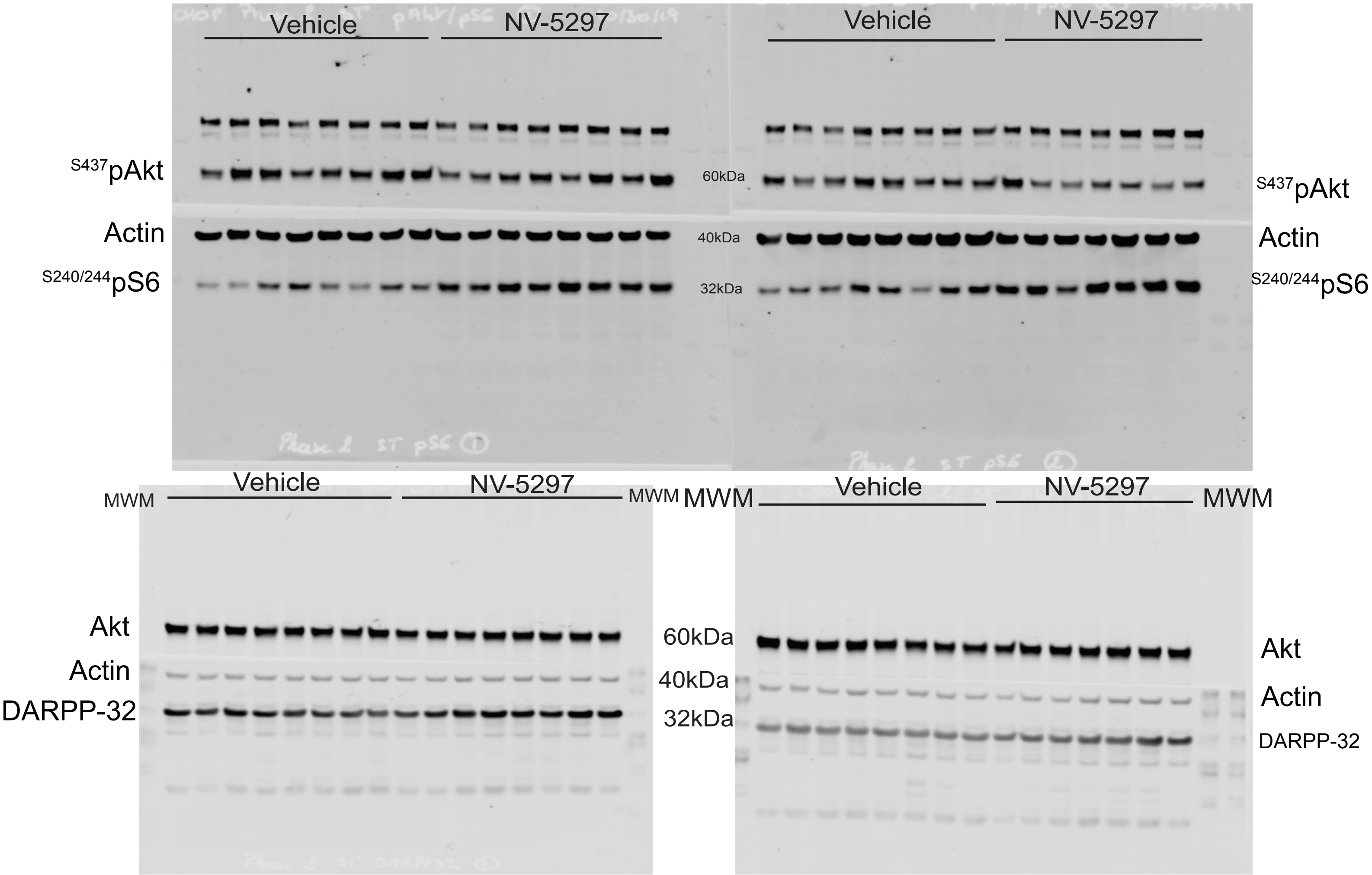

Figure 5D

MWM: Molecular weight marker
All blots imaged with LI-COR imaging system.

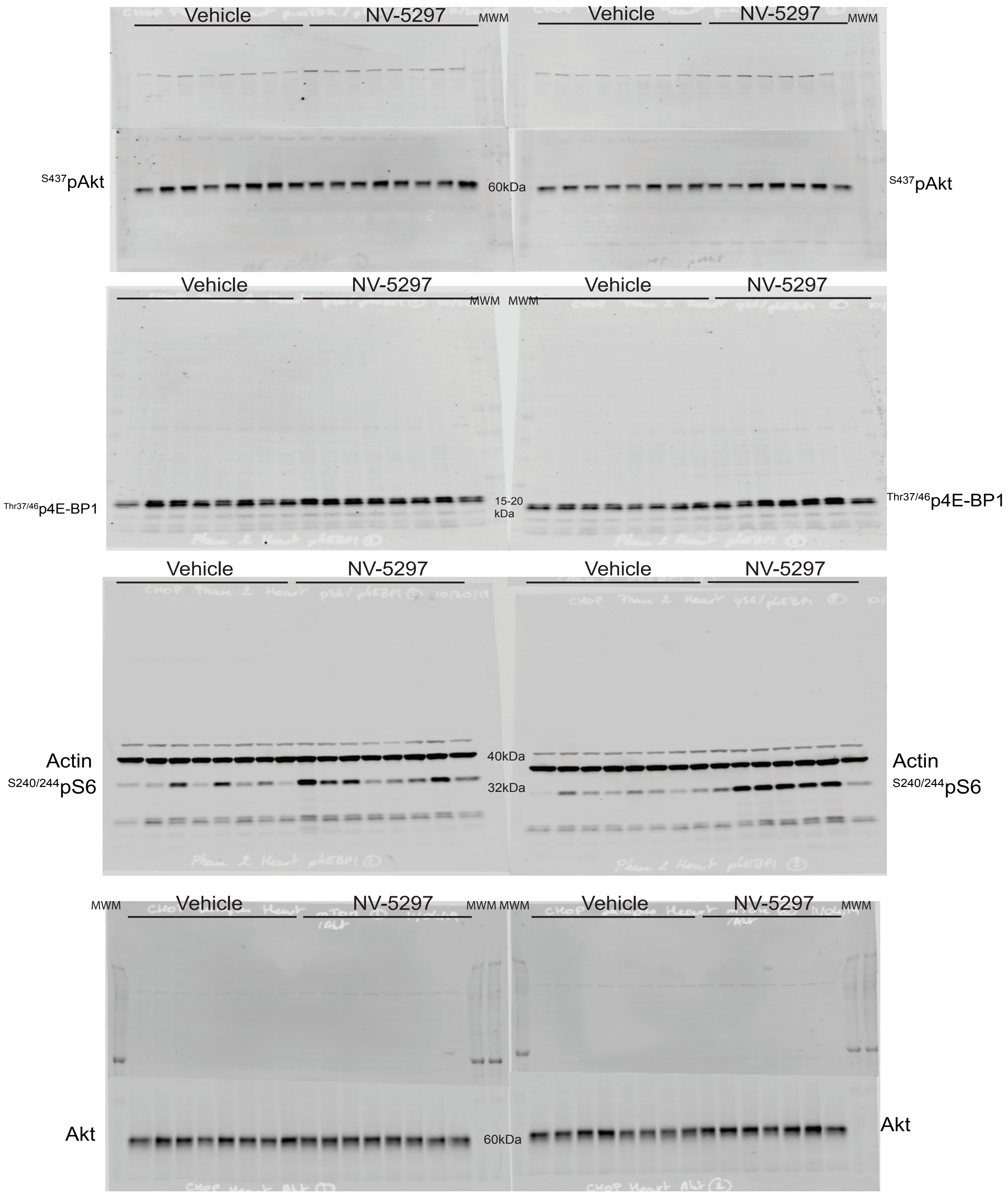

MWM: Molecular weight marker
All blots imaged with LI-COR imaging system.

Figure 9A

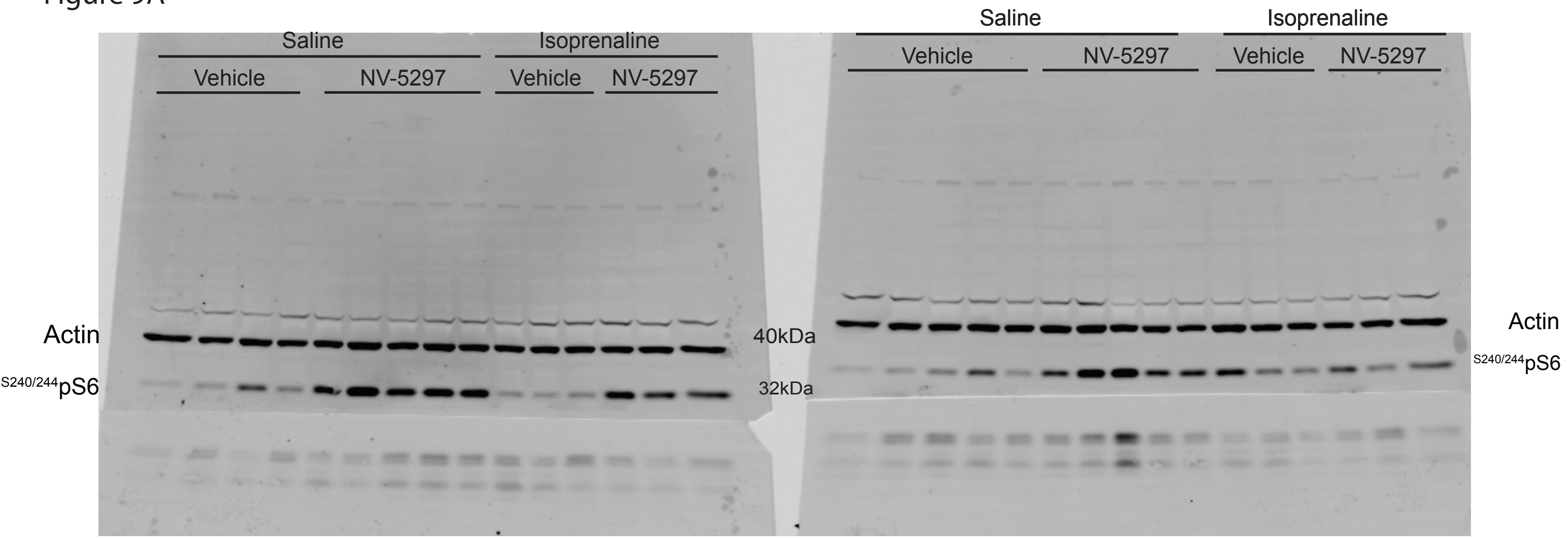

Figure 9B

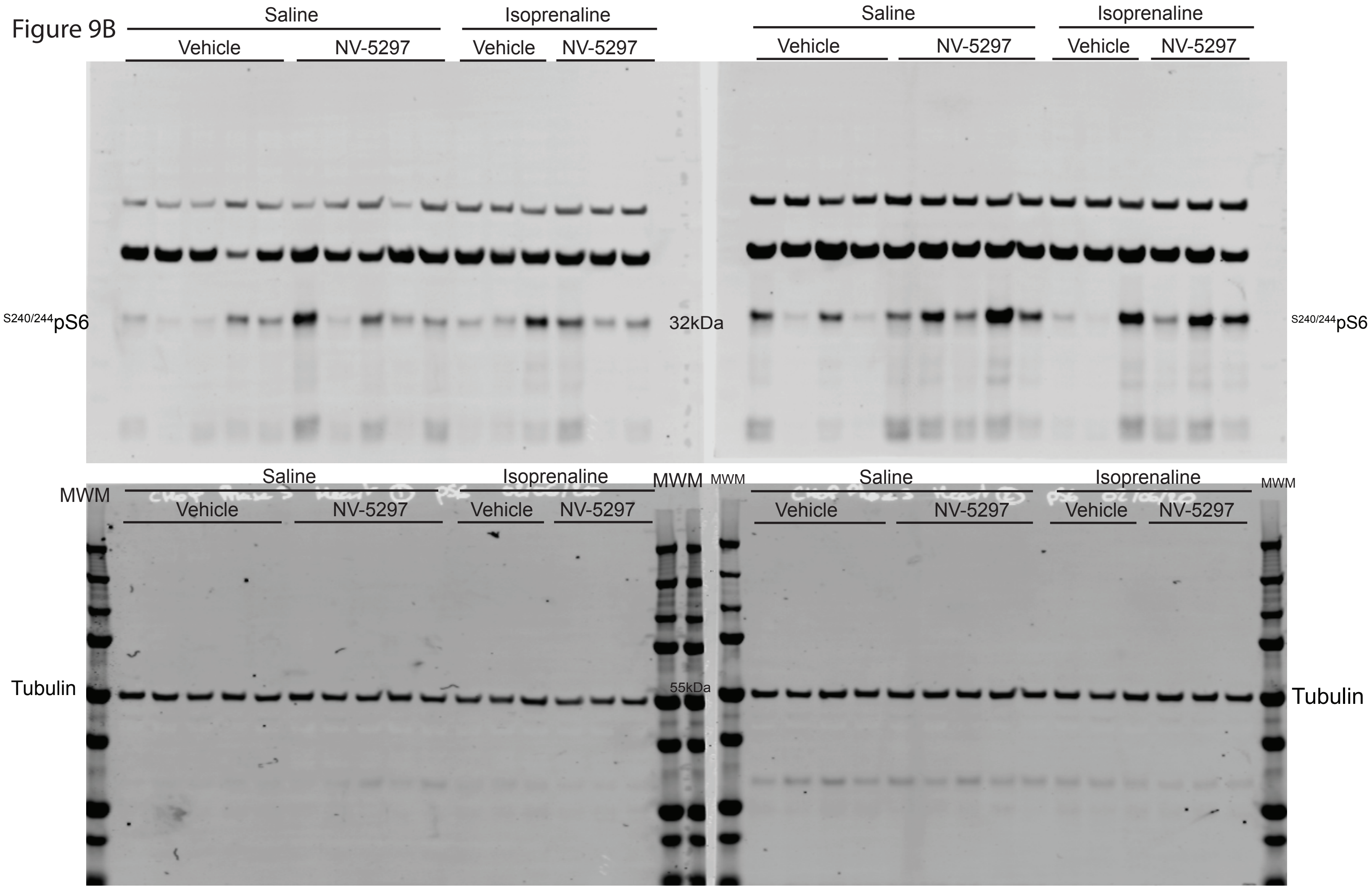

Supplementary Figure 1A

MWM: Molecular weight marker
All blots imaged with LI-COR imaging system.

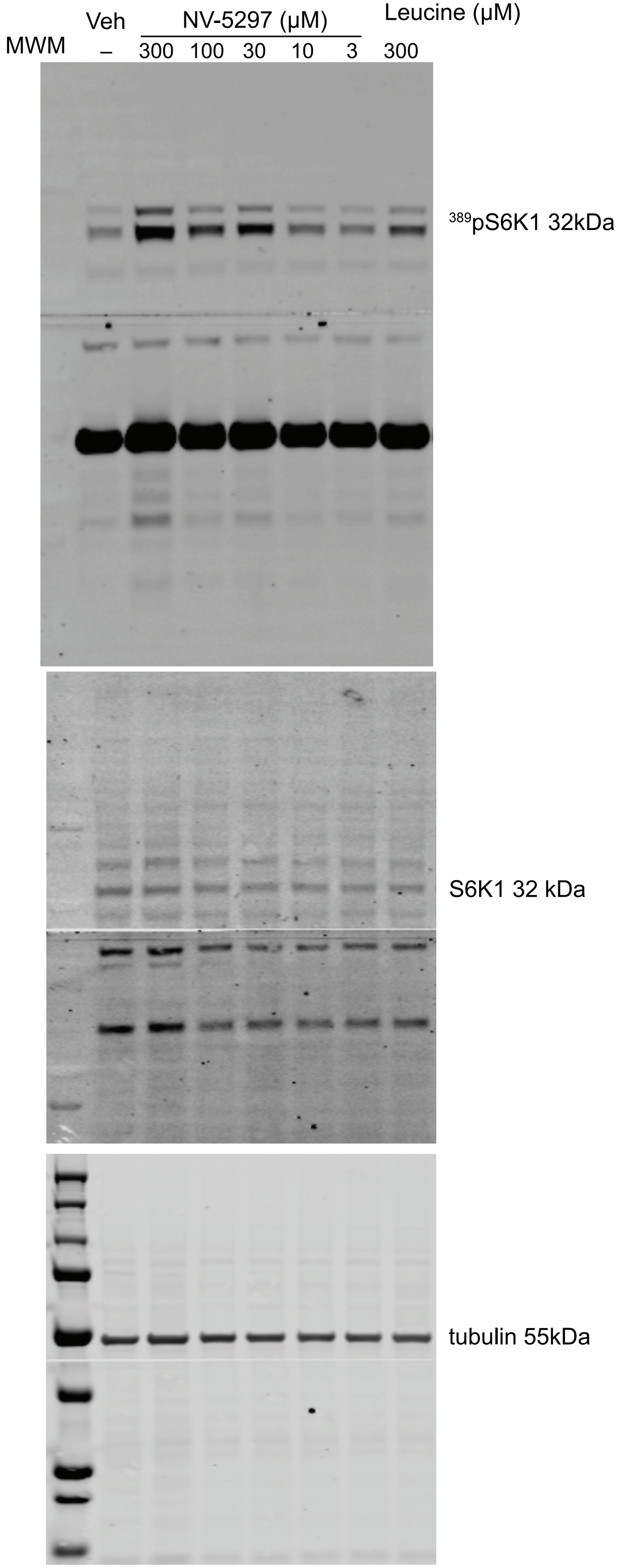

Supplementary Figure 1B

MWM: Molecular weight marker
All blots imaged with LI-COR imaging system.

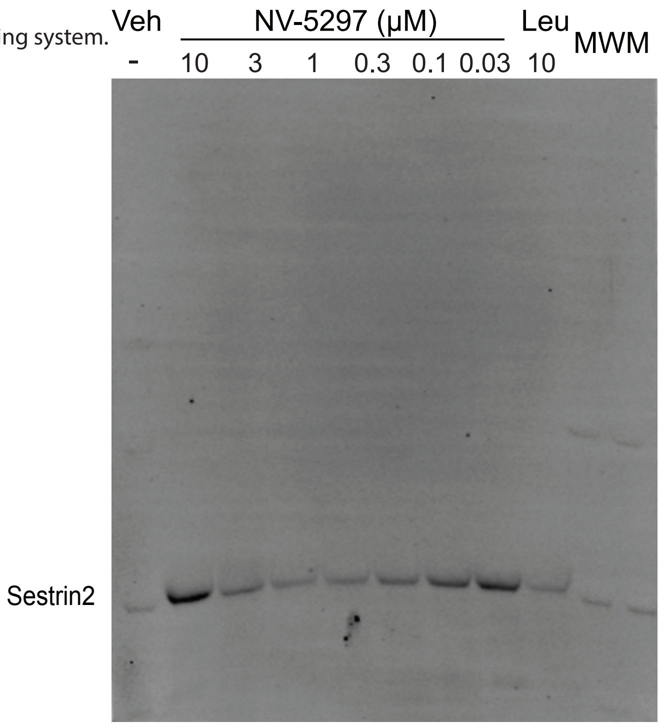

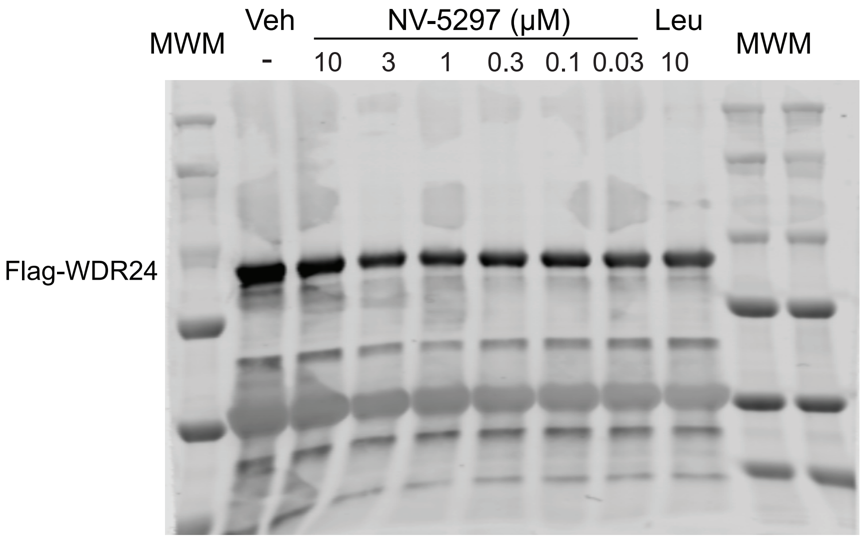

Supplementary Figure 1C

MWM: Molecular weight marker
All blots imaged with LI-COR imaging system.

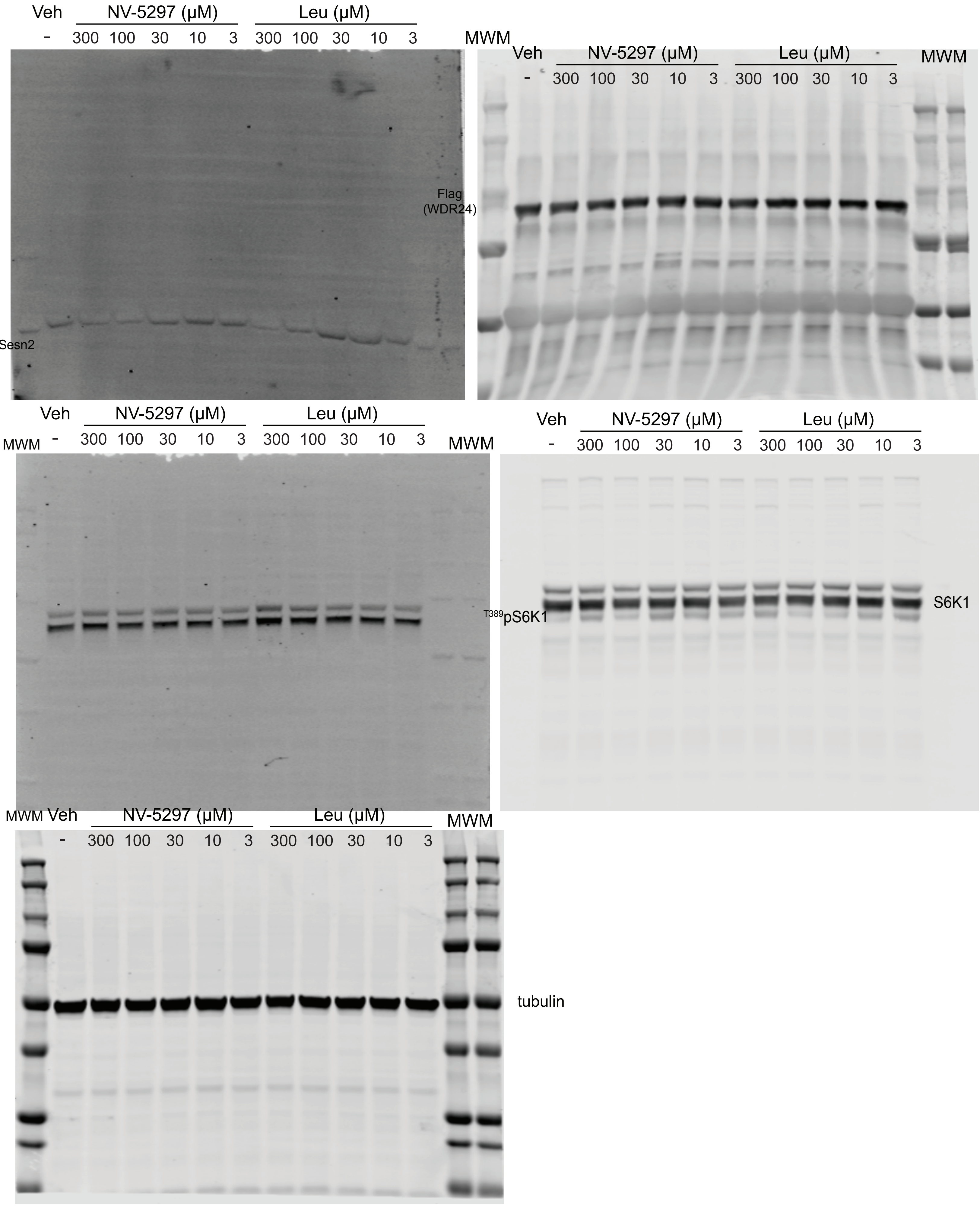

Supplementary Figure 1D

MWM: Molecular weight marker
All blots imaged with LI-COR imaging system.

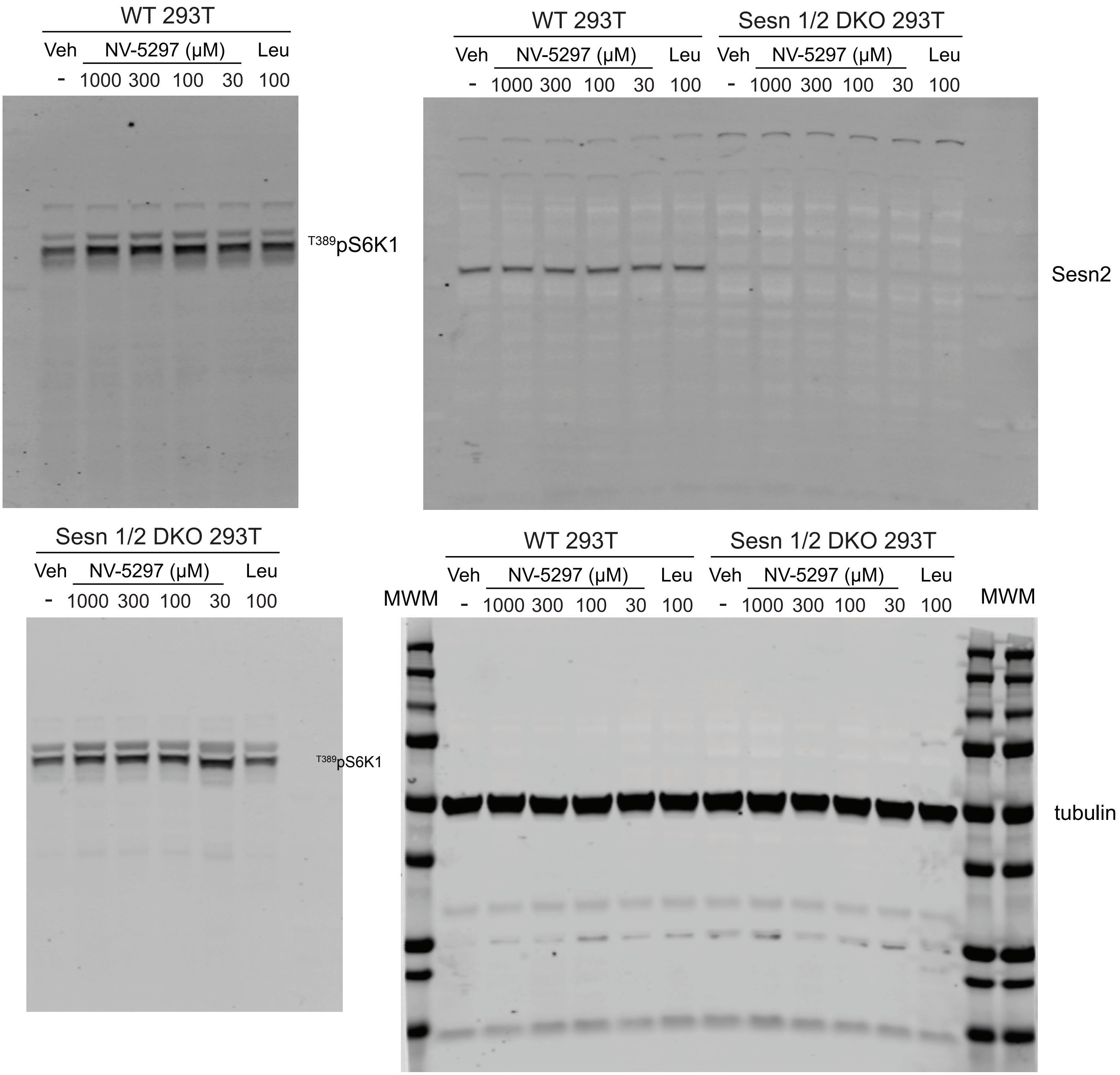

Supplementary Figure 1E

MWM: Molecular weight marker
All blots imaged with LI-COR imaging system.

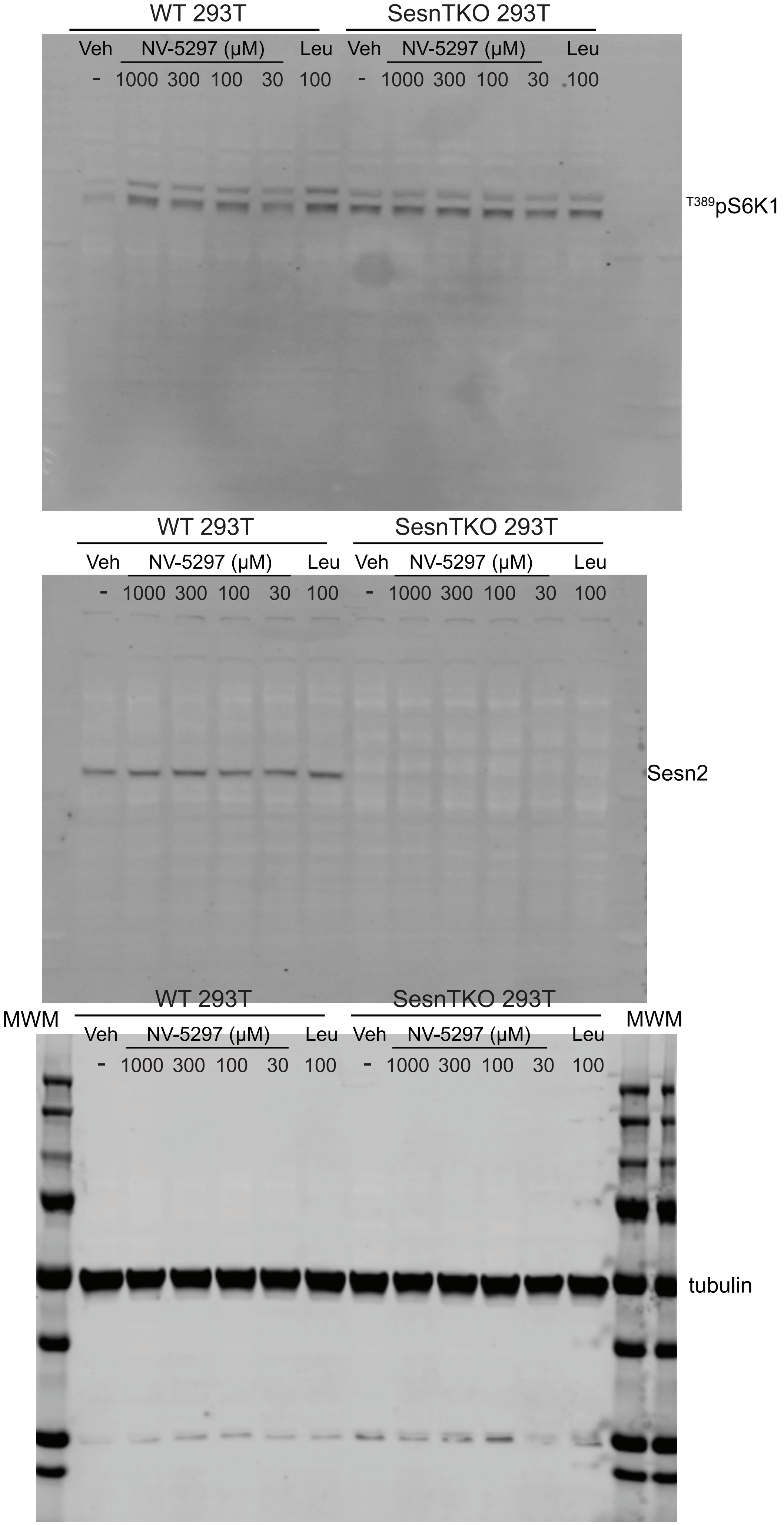

Supplementary Figure 1F

MWM: Molecular weight marker
All blots imaged with LI-COR imaging system.

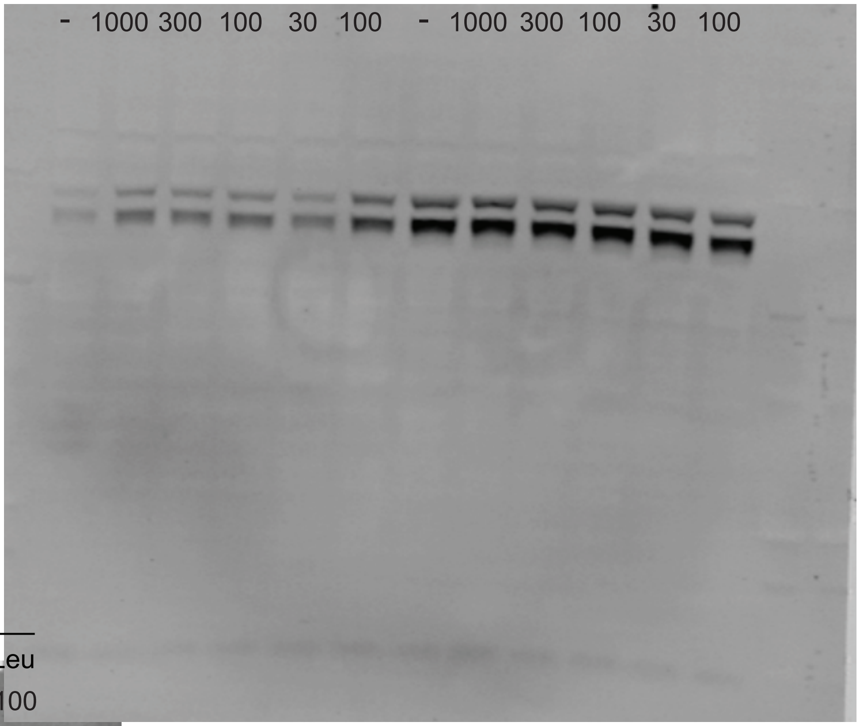

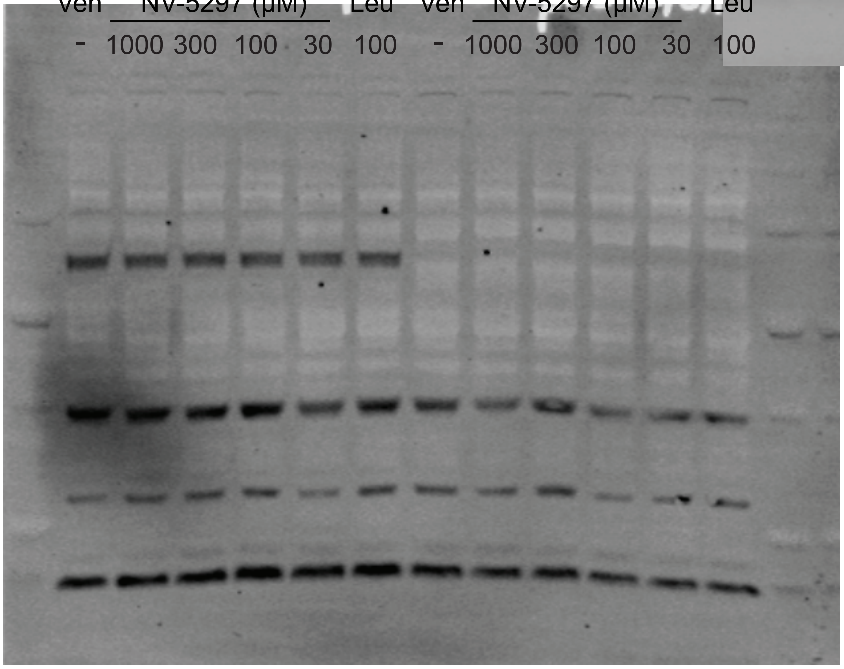

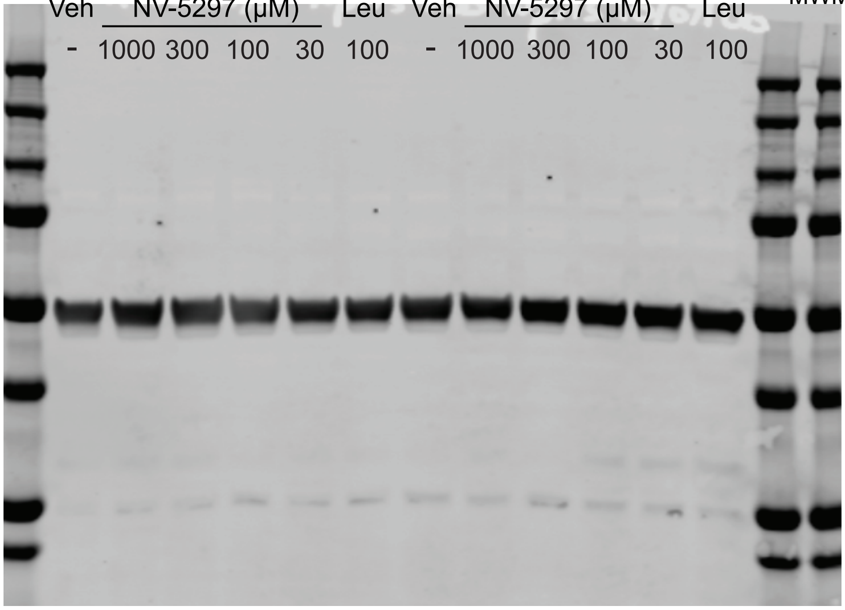

Supplementary Figure 2A

MWM: Molecular weight marker
All blots imaged with LI-COR imaging system.

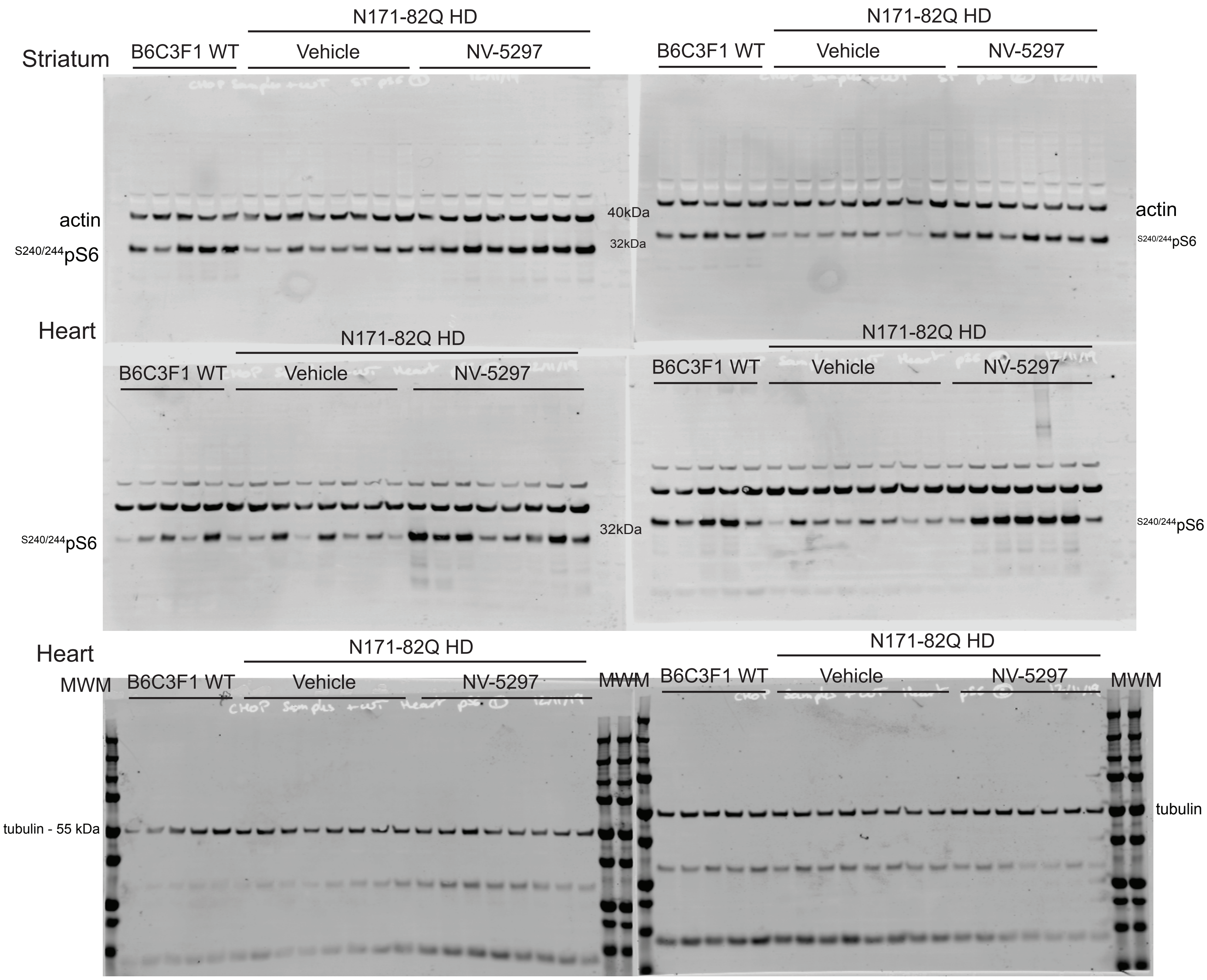

Supplement: S1 Raw images — (PDF) [file pone.0273710.s007.pdf]
