# Supplementary material for: Huntington’s disease phenotypes are improved via mTORC1 modulation by small molecule therapy
Source: PLoS One. 2022 Aug 29;17(8):e0273710. doi: 10.1371/journal.pone.0273710 (PMC9423655; doi:10.1371/journal.pone.0273710)
Supplement: Attachment — Submitted filename: Response to Reviewers.docx [file pone.0273710.s009.docx]

**Response to Reviewers**

**PONE-D-22-11888**

**Huntington’s disease phenotypes are improved via mTORC1 modulation by small molecule therapy**

Reviewer #1: Thank you to the authors - they have adequately addressed all comments
Manuscript is suitable for publication

Reviewer #2: The authors addressed all my questions in the response to reviewers. The only concern is that the answers for questions 4 and 8 were not updated in the main context. Specifically:

**1.** Please add answers to question 4 about DARPP-32 to the beginning of Line 429. (***Question 4.*** *Line 428 and Figure 3, the author measured the level of DARPP-32, but not the phosphorylated DARPP-32. Previous research shows that the function of DARPP-32 depends on phosphorylation and the phosphorylation sites. Plus, the function of DARPP-32 in striatum is controversial. Therefore, more evidence is required for the link between increased DARPP-32 and improved striatal health.)*

***Answer 1****: We added at line 431: “DARPP-32 is a specific marker of the medium spiny neurons (MSN) constituting 95% of the cell population within the striatum. DARPP-32 is a fundamental component of the dopamine-signaling cascade, and its expression is essential to the ability of dopamine to regulate the physiology of striatal neurons. Decreased DARPP-32 expression in HD starts very early in the disease and correlates with decreased neuron numbers, specifically in the striatum (Hodges et al. 2006 Human Mol Genet, Bibb et al. 2000 PNAS) and has been used in several studies including by our group (Lee et al. 2015 Neuron, Jiang et al. 2013 Human Mol Genet).” We also added on line 463: “[…], suggesting that this difference can be mainly attributed to neuronal death within the striatum.”*

2. Please justify in the main context that osmotic pump implantation in the cardiac stress experiment makes results in Figure 6 different from Figure 5, and no significant interaction of stress and cardiac stress were found so changes within the vehicle-treated groups and within the NV-5297-treated groups were focused on. (***Question*** ***8.*** ***A.*** *Figure 6E, the results of Veh-Sal without cardiac stress and NV-Sal without cardiac stress is different from Figure 5. There are changes in EF and FS between NV-5297 treatment group compared with vehicle group in Figure 5, but not here. Secondly, is there any difference between the Veh-Sal group with cardiac stress and the NV-Sal group with cardiac stress? Since there is no WT group, it is hard to tell which group is closer to “normal”.*)

***Answer 2.*** *Line 501: “The presence of this pump makes the echocardiography more challenging, justifying the difference to the baseline groups measurements along with the smaller sample size for this experiment. For this study, we found no significant interaction of stress and cardiac stress and therefore we focused on the changes within the vehicle-treated groups and within the NV-5297-treated groups.”*

3. Please explain results in Figure 6 as described in answer 8B. **(Question 8B.** Additionally, why the increases in cardiac output and heart rate are not treated as readout of increased cardiac function? In the introduction, line 56, it says that phenotypes like smaller heart size and impaired cardiac function are exacerbated by chronic ß-adrenergic agonist in HD mouse models. However, figure 6E shows increased cardiac output and heart rate in Veh-Sal group with cardiac stress. Are the results here contradicting to the previous statement?)

***Answer 3.*** *We added on line 542:* ***“****CO is calculated as the heart rate multiplied by the SV (the blood volume ejected from the left ventricle per beat). Both Veh-Iso animals and NV-Iso animals have an increase in CO compared to baseline but through different mechanisms. The increase in CO in Veh-Iso animals is due to the increase in heart rate observed to compensate for the decreased SV observed. The decrease in SV is likely due to the increase in fibrosis we observed as fibrotic tissue is stiffer and reduces muscle contractility. On the contrary, the NV-Iso animals maintain a normal heart rate but increase their CO through a more efficient contraction that led to an increase in the SV. The increase in SV is achieved through increased FS which measures ventricle diameter at the end of the diastole – ventricle diameter at the end of the systole. In turn, the increase in FS causes increased EF (percentage of blood ejected by the left ventricle by heartbeat). These changes can be observed visually with the echocardiogram in Fig 6D. Taken together, the CO increase in NV-Iso animals is done in a sustainable manner (physiologic hypertrophy), with the cardiac muscle growing to accommodate the increased workload to sustain the CO, while the Veh-Iso animals do not appropriately cope with cardiac stress (pathologic hypertrophy) and show signs of fibrosis, severe weight loss and increased mortality rate.”*
